# Protocol for a randomized controlled trial to compare bone-loading exercises with risedronate for preventing bone loss in osteopenic postmenopausal women

**DOI:** 10.1186/s12905-016-0339-x

**Published:** 2016-08-30

**Authors:** Laura D. Bilek, Nancy L. Waltman, Joan M. Lappe, Kevin A. Kupzyk, Lynn R. Mack, Diane M. Cullen, Kris Berg, Meghan Langel, Melissa Meisinger, Ashlee Portelli-Trinidad, Molly Lang

**Affiliations:** 1 College of Allied Health Professions, 984000 Nebraska Medical Center, Omaha, NE 68198-4000 USA; 2 College of Nursing, University of Nebraska Medical Center, 1230 O Street, Suite 131, Lincoln, NE 68588-0220 USA; 3 Creighton Osteoporosis Research Center, 601 North 30th, Omaha, NE 68131 USA; 4 Center for Nursing Science, University of Nebraska Medical Center, 4101 Dewey Avenue, Omaha, NE 68198-5330 USA; 5 Diabetes, Endocrinology, & Metabolism, Nebraska Medicine, Omaha, NE 68198-4130 USA; 6 Biomedical Science, Creighton University, Omaha, NE 68102 USA; 7 School of HPER, University of Nebraska at Omaha, Omaha, NE 68162-0216 USA; 8 Heartland Osteoporosis Research Study, College of Allied Health Professions, 984000 Nebraska Medical Center, Omaha, NE 68198-4000 USA

**Keywords:** Osteoporosis, Low bone mass, Postmenopausal women, Calcium and vitamin D, Risedronate, Bone-loading exercises, Bone mineral density, Bone structure

## Abstract

**Background:**

In the United States, over 34 million American post-menopausal women have low bone mass (osteopenia) which increases their risk of osteoporosis and fractures. Calcium, vitamin D and exercise are recommended for prevention of osteoporosis, and bisphosphonates (BPs) are prescribed in women with osteoporosis. BPs may also be prescribed for women with low bone mass, but are more controversial due to the potential for adverse effects with long-term use. A bone loading exercise program (high-impact weight bearing and resistance training) promotes bone strength by preserving bone mineral density (BMD), improving bone structure, and by promoting bone formation at sites of mechanical stress.

**Methods/Design:**

The sample for this study will be 309 women with low bone mass who are within 5 years post-menopause. Subjects are stratified by exercise history (≥2 high intensity exercise sessions per week; < 2 sessions per week) and randomized to a control or one of two treatment groups: 1) calcium + vitamin D (CaD) alone (Control); 2) a BP plus CaD (Risedronate); or 3) a bone loading exercise program plus CaD (Exercise). After 12 months of treatment, changes in bone structure, BMD, and bone turnover will be compared in the 3 groups. Primary outcomes for the study are bone structure measures (Bone Strength Index [BSI] at the tibia and Hip Structural Analysis [HSA] scores). Secondary outcomes are BMD at the hip and spine and serum biomarkers of bone formation (alkaline phosphase, AlkphaseB) and resorption (Serum N-terminal telopeptide, NTx). Our central hypothesis is that improvements in bone strength will be greater in subjects randomized to the Exercise group compared to subjects in either Control or Risedronate groups.

**Discussion:**

Our research aims to decrease the risk of osteoporotic fractures by improving bone strength in women with low bone mass (pre-osteoporotic) during their first 5 years’ post-menopause, a time of rapid and significant bone loss. Results of this study could be used in developing a clinical management pathway for women with low bone mass at their peak period of bone loss that would involve lifestyle modifications such as exercises prior to medications such as BPs.

**Trial registration:**

Clinicaltrials.gov NCT02186600. Initial registration: 7/7/2014.

**Electronic supplementary material:**

The online version of this article (doi:10.1186/s12905-016-0339-x) contains supplementary material, which is available to authorized users.

## Background

Osteoporosis is a disease of fragile bones (decreased bone strength) and low bone mineral density (BMD) that is frequently complicated by fractures. According to the National Osteoporosis Foundation (NOF), 9.9 million Americans have osteoporosis, an additional 43.1 million have low bone mass or osteopenia, and 80 % of Americans with osteoporosis or low bone mass are women [1]. Osteoporotic fractures are an enormous medical and personal burden for these women [2, 3]. The 1-year mortality rate after an osteoporotic hip fracture is approximately 20 % [4], and increased mortality from hip fractures extends to 10 years after the fracture [5]. The economic impact of osteoporotic fractures is large and growing [6]. From 2000 to 2011, there were 4.9 million hospitalizations for these fractures, and annual costs for hospitalization was greater than 5.1 billion dollars. The number of hospitalizations and hospital costs were greater for osteoporosis than for myocardial infarction, stroke, or breast cancer patients [6]. Our research aims to decrease the risk of osteoporotic fractures by improving bone strength and preserving BMD in women with low bone mass during their first 5 years’ post-menopause, a time of rapid and significant bone loss [7].

This paper presents the study protocol for a prospective, stratified, randomized controlled trial that is federally–funded and currently in progress. This study compares changes after 12 months in bone structure, BMD, and bone turnover in post-menopausal women with low bone mass stratified by exercise history and randomized to one control or two treatment groups (*n* =103 per group): 1) calcium + vitamin D (CaD) alone (Control); 2) A bisphosphonate (BP) plus CaD (Risedronate); or 3) a bone loading exercise program plus CaD (Exercise). Improving bone strength and preventing further loss of bone is critical in postmenopausal women with low bone mass as it is difficult to build significant bone without the influence of estrogen [8]. Preventative treatments are especially important during the first 5 years’ post-menopause because women are more likely to have bone loss during this time. Recker et al. (2000) reported that women lose more than 5 % of spine BMD in the 5 years after the last menses [8]. Calcium and vitamin D supplements (CaD), exercise, and BPs are all used to address bone health in women. There is strong evidence that adequate CaD through dietary intake and/or supplementation is necessary to maintain bone health [9, 10]. Although CaD is more effective than no treatment, it frequently is not enough to prevent progression of bone loss or to prevent osteoporosis. BPs such as risedronate are the medications of choice for treatment of osteoporosis [3], and studies have reported that use of risedronate attenuates the bone loss associated with menopause in osteoporotic women [11].

The best predictor of fracture is bone strength, and strength is determined by bone structure as well as BMD. While BPs are effective in improving bone density by inhibiting bone resorption, findings on the effectiveness of BPs in improving bone structure are inconsistent [12, 13]. In addition, after 5 years of BPs, rare safety concerns such as atypical femur fractures become more common [14, 15]. Researchers have suggested that long-term use of BPs can result in severe suppression of bone turnover with significant accumulation of micro-damage in bone [16]. This micro-damage can lead to increased risk of atypical hip and femur fractures [17–19]. Due to concerns about their long-term use, providers frequently discontinue prescriptions for BPs after 3 to 5 years of treatment in patients who are at modest risk of fracture [3, 20, 21].

Exercise programs combining resistance and high-impact weight bearing exercises can improve bone structure and increase bone formation at sites of mechanical stress [22] as well as maintain BMD in postmenopausal women. Participation in exercises improves the ratio of bone formation to resorption and improves bone structure by stimulating increased formation of trabecular and cortical structure at sites of stress, increasing thickness of cortical tissue, improving trabecular bone geometry and microarchitecture, and reorganizing bone collagen. Collagen reorganization as a result of exercise maintains bone strength, even when BMD is decreased by as much as 10 % [23]. Participation in exercises is not associated with severe suppression of bone turnover, and furthermore, exercise is associated with many other positive effects for postmenopausal women, including increased insulin sensitivity, improved functional ability and decreased risk of falls and depression [22]. If an effective exercise program could substitute for or delay the use of BPs in post-menopausal women with low bone mass, not only would bone health improve throughout the lifespan, but women would also benefit from the many other positive effects of exercise.

Our long-term goal is to contribute to the development of clinical practice guidelines for the promotion of bone health in postmenopausal women with low bone mass. Clinical management pathways for women who are at risk for other chronic illnesses such as hypertension or diabetes include trials of lifestyle modifications prior to prescriptions for medications. With the potential for adverse effects with long-term use of BPs, the significant consequences of progression to osteoporosis, and the potential bone strength benefits of exercise, studies should be conducted to determine if prescriptions for exercises are warranted prior to prescriptions for medications.

## Methods/Design

### Ethics approval

This study was approved by the Institutional Review Board (IRB) of the University of Nebraska Medical Center. Written consent was obtained from participants at the time of screening and again at the time of enrollment using two separate documents. Subjects receive both the screening consent and main study consent documents for review prior to their screening visit. Careful and thorough explanation are utilized in obtainment of consent at screening and enrollment to ensure participant comprehension.

### Study aims

- Aim 1. To compare Control, Risedronate, and Exercise group subjects on changes in bone structure at the tibia (bone strength index [BSI]: pQCT) and hip (hip structural analysis [HAS]: DXA) at 6 and 12 months.
- Aim 2. To compare Control, Risedronate, and Exercise group subjects on changes in BMD at the total hip, femoral neck, and spine (DXA) at 6 and 12 months.
- Aim 3: To compare Control, Risedronate, and Exercise group subjects on changes in bone formation and resorption at 6 and 12 months. (formation: AlkphaseB, resorption: Serum NTx).
- Aim 4: To explore relationships between adherence to exercise (% sessions attended), adherence to risedronate (% pills taken), and changes in bone structure at the tibia (BSI) and hip (HSA).

### Research design

This study uses a prospective stratified (by exercise history), randomized, 3-group repeated measures experimental design with three major data collection points (baseline, 6, and 12 months). All subjects are permitted to continue their usual physical activity during the study. However, prior to randomization, subjects are stratified by exercise history (≥2 high intensity exercise sessions per week; < 2 sessions per week) to ensure equal distribution among the three groups. Exercise history will be categorized using the International Physical Activity Questionnaire (IPAQ), an instrument used for cross-national monitoring of physical activity and inactivity. Subjects are then randomized to a control or one of two treatment groups (*n* =103 per group): 1) calcium + vitamin D (CaD) alone (Control); 2) BP plus CaD (Risedronate); and 3) a bone loading exercise program plus CaD (Exercise). Treatments are 12 months in duration. Primary outcomes for the study are bone structure measures (Bone Strength Index [BSI] at the tibia [pQCT] and Hip Structural Analysis [HSA] scores). Secondary outcomes are BMD at the hip and spine (DXA) and serum biomarkers of bone formation (AlkphaseB) and bone resorption (Serum NTx). Our central hypothesis is that improvements in bone strength will be greater in subjects randomized to the Exercise group compared to subjects in either the Control or Risedronate groups.

### Sample size

A power analysis for sample size was conducted based on the primary aim of the study.

Our hypothesis states that at 12 months, there will be significantly greater improvements in BSI at the 4 % tibial site in subjects in the Exercise group compared to subjects in the Control or Risedronate groups. Assuming a significance level of 0.05, three time points, and an estimate of 0.5 for the correlation between time points, 164 participants (82 per group) would be needed to have 80 % power. Since this study will involve three groups, a total of *N* = 246 will be needed (82*3), which will be randomized and evenly allocated across three groups. In order to account for a realistic amount of attrition (20 %), 309 participants will be recruited for this study so that at least 246 should be retained if 20 % of participants do not complete the study. Kemmler et al. (2002) observed a 15 % attrition rate over 14 months in an exercise study, so we expect there to be well under 20 % attrition in this intent-to-treat analysis study [24].

### Recruitment and eligibility

Recruitment of subjects will focus on the Omaha, NE and Lincoln, NE areas as well as Council Bluffs, IA. Women of all races and ethnicities are encouraged to participate in the study. Subjects will be recruited through physician and/or practitioner referral; community presentations (especially when held in conjunction with ongoing corporate and community wellness programs); personal contact; television, radio, and newspaper ads; distributing flyers, postcard and letter mailings; and generation of a website and Facebook page. Recruitment strategies will target the specific women who meet the criteria for our study (e.g., within 5 years’ post-menopause, diagnosis of low bone mass). All recruitment materials will have prior approval from the IRB. Inclusion and exclusion criteria for subject eligibility are summarized in Table 1.

**Table 1:** Inclusion and Exclusion criteria for subjects in study

Inclusion Criteria	Exclusion Criteria
• Women in first 5 years of menopause	• BMO T Score < -2.5 at hip or spine (osteoporosis)
• BMD T score between -1.0 and -2.49 at total hip or L1L4 spine (osteoporosis)	• Increased hip and major fracture based on FRAX score
• 19 years of age or older	• Bisphosphonates in last 6 months
• Health care provider’s permission to be in study	• Currently on estrogen, tamoxifen, Aromatase Inhibitors, others
	• Weight > 300 pounds
	• Serum Vitamin D <10 ng/ml or >100 ng/ml
	• Any conditions that prohibit optimal CaD, risedronate, or exercise

### Screening and enrollment

Subjects complete the prescreening forms online or during a phone contact interview. Prescreening forms include evaluation of inclusion/exclusion criteria, and the Physical Activity Readiness Questionnaire (PARQ). Subjects who meet study criteria during the prescreening interview are scheduled for BMD (DXA) testing at the hip and spine and blood work (serum 25[OH]D, parathyroid hormone [PTH], calcium, creatinine, and thyroid stimulating hormone [TSH]) to screen for eligibility for the study.

**Fig. 1:**
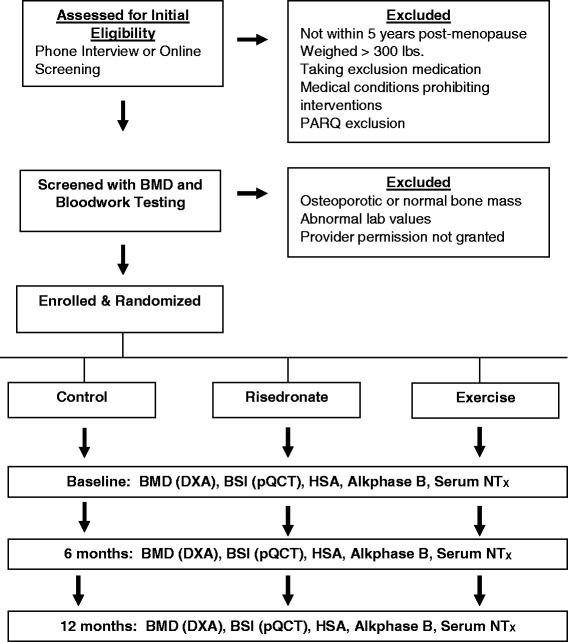
Experimental design and flow chart

Prior to BMD testing and blood work, and after the consent process for screening, medical and physical activity histories are collected. If BMD testing identifies a subject as having low bone mass, the subject is at low risk of fracture via the Fracture Risk Assessment tool (FRAX), all laboratory tests are within normal limits, and the individual would like to participate, a letter is sent to the subject’s primary care provider requesting approval for the subject to participate in the main study. After consenting to participate in the study, bone structure at the tibia is tested using peripheral Quantitative Computed Tomography (pQCT) (Stratec XCT 3000). Each subject is stratified by exercise history and randomized to one of the three groups, using computer-generated random numbers allocated by our statistician. The subject and research team member learn the subject’s group assignment when the subject opens a sealed, opaque envelope after consenting to enrollment. See Fig. 1.

### Setting

All data collection for subjects, including bone structure and BMD testing, is performed at the Creighton University Osteoporosis Research Center (CORC), located in Omaha, NE. Bone structure (HSA) at the hip and BMD testing is performed using the OASIS-APEX Workstation with APEX 4.0.X software and the Hologic DXA. Hip Structural Analysis (HSA) measures are obtained from DXA results. Critical issues in obtaining DXA and HSA results are the importance of using the same densitometer over time and proper positioning of the hip. All subjects use the same Hologic DXA machine throughout the study. The radiology technician at the CORC is certified in densitometry testing and experienced in positioning the hip to provide for the most accurate BMD and HSA results.

In addition to the DXA testing at baseline, 6, and 12 months, all women obtain pQCT (Stratec XCT 3000) testing of the tibia at the CORC. All analysis of pQCT results is performed by Dr. Diane Cullen, our co-investigator at Creighton University, who has been researching bone structure and pQCT testing and publishing results of her studies for over 18 years.

All blood is drawn by our research team members at the CORC at baseline, 6, and 12 months at the time women report for their BMD testing. Team members drawing blood are certified in phlebotomy training and/or have many years of experience with phlebotomy. Blood is transported from the CORC to the Clinical Research Center (CRC) at UNMC or to laboratory refrigerators in the College of Allied Health. Certified technicians at the CRC analyze all blood work except for Serum NTx and AlkphaseB. Serum NTx and AlkphaseB testing are performed by Dr. Laura Bilek and Molly Lang, MS, and analyzed by ELISA.

### Intervention components

#### Calcium and Vitamin D (Control group)

The National Osteoporosis Foundation (NOF) recommends that women over age 50 consume 1200 mg of Ca and 800–1000 IU of vitamin D_3_ per day [3]. To ensure subjects are obtaining sufficient daily calcium, information on their dietary intakes of calcium and use of supplements are obtained at baseline. Dietary intakes of calcium are calculated using the National Osteoporosis Foundation (NOF) Calcium Intake Estimate. Based on results, calcium citrate supplements are prescribed as needed so that subject’s total intake (dietary and supplemental) is ~1200 mg. Vitamin D levels may be low in subjects as low levels have been documented in 68 % of 1179 post-menopausal, rural American women residing in northern climates, and in 52 % of 1536 post-menopausal North American women taking medications for osteoporosis [25, 26].

The protocol in this 12-month study for confirming that women with low bone mass obtain sufficient vitamin D, despite seasonal variations, was developed by the co-investigator, Dr. Joan Lappe. Dr. Lappe has performed extensive research in both Ca and D [26]. Subjects are prescribed doses of vitamin D based on their serum 25(OH) D levels at baseline, with the goal of subjects reaching a serum level of at least 30 ng/ml [27]. If subjects are currently taking vitamin D supplements, they will be encouraged to continue taking their supplements. In addition to their current intake of supplements, subjects who have serum D levels at 30 ng/ml or greater will be prescribed 1000 IU daily; subjects with levels of 20–29 ng/ml will be prescribed 2000 IU daily; and subjects with levels of 10–19 ng/ml will be prescribed 3000 IU daily. Subject’s combined intake of vitamin D supplements (usual intake as well supplements prescribed for the study) will be limited to 4000 IU of vitamin D daily. Subjects receiving ≥ 3000 IU daily will have their serum levels rechecked at 3 months. All other subjects will have follow-up serum levels at 6 months. Time periods for assessing serum vitamin D were chosen because studies have reported that with daily dosing of D, it may take three months for plateau serum levels to be reached. Protocols for ensuring sufficient calcium and vitamin D and prescribing supplements pertain to all three groups.

#### Medication calendar cards

CaD tablets will be supplied in Medication Calendar cards prepared by the Nebraska Medicine outpatient pharmacy and distributed by mail quarterly. Tablets will be secured in individual bubbles on the cards. Subjects will be instructed to take the tablet for the corresponding day and time, and if they do not take it for any reason, to leave it in the bubble and move to the next time. All cards with any remaining tablets will be collected by a team member or returned by postage-paid mail after each 4-week period. Remaining tablets will be counted to determine adherence to CaD. This procedure has worked well in numerous previous studies of adherence to medications, with less than 5 % missing pill count data [28].

#### Risedronate group

Subjects randomized to the Risedronate group will take the prescribed CaD daily and a 150 mg tablet of oral risedronate once every 4 weeks for 12 months. Risedronate is a pyridinyl bisphosphonate with potent anti-resorptive activity which was approved at the 150 mg monthly dose for the prevention and treatment of post-menopausal osteoporosis. BPs such as risedronate prevent bone loss by inhibiting the activity of osteoclasts, thus decreasing resorption or loss of bone [11, 29].

#### Bone-loading exercise program

Recommendations for prevention of osteoporosis as well as other chronic diseases suggest participation in both resistance training and weight bearing activities with high bone-loading forces 3–5 times per week [29, 30]. Subjects randomized to the Exercise group will take the prescribed CaD daily and also participate in 12 months of a bone-loading exercise program three times weekly at local, partner, community-based fitness centers. Exercises developed for this study include high-impact, weight-bearing exercises consisting of jogging using a weighted vest and resistance exercises. One Repetition Maximum (1RM) testing will be used to evaluate if resistance provided by weight machines is at a therapeutic level. Settings or weight loads for machines will be increased based on: a) subject’s performance during the 1RM testing; and b) performance relative to volitional fatigue. The goal is to progress to a resistance of 70 to 85 % of 1 RM or which results in volitional fatigue at 8–12 repetitions. Each session will include warm-up and cool-down exercises consisting of 5 min of slow walking.

To promote optimal performance and adherence, subjects will be provided a detailed training journal/exercise log. It will include detailed written and visual supportive instructions for each exercise including specific prescribed exercise session parameters to guide the subjects. All exercises will be performed under the direction of the research exercise trainer (RET) and onsite exercise trainers (ETs) employed by the community fitness center. After initial in-person training sessions by the RET and on-site ET, on-site ETs will communicate with each exercise subject every 2 weeks during the 12-month study to provide direction on exercise progression. Continued RET oversight of all ET activities will provide education, training, and interaction with subjects. Additional file 1. The Exercise Loading Program describes the detailed exercise protocol and progression [See Additional file 1].

#### Procedures for intervention fidelity

Low adherence rates to exercises, risedronate, and CaD would impact fidelity of intervention components and limit generalizability of study results. Evidence suggests that exercise sessions 3 times weekly are critical for bone-loading [31]. Studies by Knobf and others [32] have reported that post-menopausal women are able to adhere to 3 times weekly exercises. In a previous study, adherence to exercises was significantly correlated with task and barrier self-efficacy (confidence in knowledge of exercise, *r* = .22, *p* < .05; confidence in overcoming barriers to exercise, *r* = .49, *p* < .05) [29]. According to Bandura (1997), task self-efficacy is subjects’ confidence in their ability to perform interventions, such as exercises, and barrier self-efficacy is subjects’ confidence in their ability to overcome barriers to interventions. In the proposed study, we will promote task and barrier self-efficacy through subject education, goal setting, standardized barrier reduction strategies, and by providing graphic feedback on goal achievements [33].

Feedback sheets will be provided to subjects to demonstrate progress over time in adherence to study treatments (CaD, risedronate, exercises) and in changes or preservation of BMD. Team members (research coordinator, RET, on-site ETs) will be oriented to use of all strategies for promoting self-efficacy and adherence. Scripts will be provided for assisting with goal-setting and reducing barriers to promote standardization across sites. Strategies for reducing barriers to adherence to exercises will be tailored to subjects based on barriers identified on a Barriers Interference instrument. This instrument was developed by Dr. Laura Rogers who has numerous publications on exercise adherence and barriers to adherence [34, 35].

### Study outcomes

The best predictor of fracture risk is bone strength. This study will include three measures of bone status because the strength of bone depends not only on the amount of bone present (BMD) but also on bone structure or geometry and on positive bone turnover rates (ratio of bone formation to resorption) [36]. The primary outcome will be bone structure at the tibia and hip, and secondary outcomes are BMD and bone turnover. Assessors of these outcomes will be blinded to subject group. Repeated measures of primary and secondary outcome variables will be conducted at baseline, 6 and 12 months to better understand the response to treatment components with a maximum difference expected at 12 months.

#### Bone mineral density

The gold standard for diagnosis of osteopenia and osteoporosis is BMD obtained from DXA testing [37]. DXA results provide the most accurate measure of BMD and the preferred sites for diagnosis of osteoporosis are BMD measures at the L1L4 spine, total hip, and femoral neck [37].

#### Bone structure

Bone structure refers to the cortical and trabecular network of bone that is vital for maintaining maximum bone strength [38]. In the proposed study, bone structure measures include Bone Strength Index (BSI) at the tibia and Hip Structural Analysis (HSA) [39]. BSI will be measured at the 4 %, and 66 % tibial sites using pQCT. BSI is based upon the total bone area, tissue density, bone porosity, and distribution or shape (moment of inertia) at that bone site and has been found to be a strong predictor of the torsion and bending strength of bones [12]. HSA scores are calculated using software and results of DXA testing. HSA refers to the distribution of bone mass at the hip. The specific data collected includes the cross-sectional area [CSA] occupied by bone mineral, an index of bone strength against compression; section modulus [Z, mm3], an index of bone strength against bending; and centroid [mm], the distance from the center of the mass to the periphery [39].

#### Bone turnover

Bone turnover is the process of removing old bone (resorption by osteoclasts) and replacing it with new bone (formation by osteoblasts) [40]. In this study, resorption will be measured by Serum NTx (nmol/BCE/L) and formation by AlkphaseB (U/C) at baseline, 6, and 12 months.

### Data collection and entry

Data are collected on subjects at baseline, 3, 6, and 12 months. Besides subject and team member reports and pill counts, data collected include results of radiology testing (BMD and bone structure) and laboratory testing (serum vitamin D, calcium, PTH, TSH, creatinine, AlkphaseB, serum NTx). All data are entered by subjects or team members directly into the Research Electronic Data Capture (Redcap) database using iPads. The datasets analyzed during the current study will be available from the corresponding author on reasonable request. 

The research coordinator is assigned overall responsibility for data collection and data entry for all subjects randomized to the Control group and to the Risedronate group at baseline, 3, 6, and 12 months. The RET is assigned overall responsibility for data collection and data entry into Redcap for all subjects in the Exercise group. Data quality is promoted using double data entry and range checks for data values. Study variables, instruments, outcome measures, and data collection time points are included in Table 2.

**Table 2:** Study variables, instruments, and rationale for use

Measure	Purpose	Baseline	3 months	6 months	12 months
Demographic profile and health history [3]	Demographic data and medical history to describe sample and identify potential covariates	X			
Physical activity readiness questionnaire (PARQ)	Predicts the need for medical clearance for initiating exercise	X			
Calcium, PTH, 25(OH)D, TSH, Creatinine (Cr) [27]	To identify abnormalities which exclude participation, and serum Vit D informs prescription for dosage of Vit D	X	X^a^	X^a^	
National Osteoporosis Foundation Calcium Intake Estimate	Calculates dietary intake of calcium based on servings of calcium-rich food per day	X			
Fracture risk Assessment tool (FRAX) [3]	Estimates 10 year risk of osteoporotic fracture	X			
Incidence of Fractures Documentation Form	Incidence, type, cause, treatment of fracture	X	X	X	X
International Physical Activity Questionnaire (IPAQ)	Data used at baseline to stratify subjects by exercise history prior to randomization to group	X		X	X
Adherence to CaD, risedronate, exercises [29]	Documentation of unused tablets in individual bubbles of returned medication cards or prescribed exercise sessions attended	X	X	X	X
Task and Barrier Self-Efficacy Scales [33–35]	Data collected on subject confidence in ability to perform interventions (CaD, exercises, risedronate)	X	X	X	X
One Repetition Maximum (1 RM)	Maximum weight lifted once through full range of motion (ROM) to measure muscle strength	X	X		
Barriers Interference [34, 35]	Barriers to exercise adherence perceived by subjects	X	X	X	X
Dual-Energy X-Ray Absorptiometry (DXA) [37]	BMD at total hip, femoral neck, and spine	X		X	X
Peripheral Quantitative Computed Tomography (pQCT) [12]	Bone Strength Index (BSI) at 4 % & 66 % sites non- dominant tibia, Stratec XCT 3000	X		X	X
Serum Biomarkers of Bone Turnover [36, 40]	AlkphaseB (U/C) & Serum NTX (nmol BCE/L), assesses rate of bone formation and resorption	X		X	X
Hip Structural Analysis (OASIS-APEX Workstation + APEX 4.0.X software and Hologic DX44) [39, 47]	HSA of femoral neck, femoral shaft, and intertrochanteric is a measure of hip structure calculated from the DXA result	X		X	X

^a^25(OH)D testing only

### Data analysis

Before proceeding with analysis, a careful descriptive study will be conducted to evaluate distributional assumptions of all variables. Primary analyses will be conducted consistent with the intent-to-treat paradigm, with each participant’s data analyzed according to group assignment. The hypotheses for aim 1 state that: *At 12 months, there will be significantly greater improvements in BSI at the 4 % tibial site and significantly greater improvements in bone mass distribution (HSA; centroid, mm) at the femoral neck in Exercise subjects compared to subjects in either Risedronate or Control groups.* These hypotheses will be tested via RM-ANOVA models. A significant time group interaction would indicate that the three groups being compared in each model have significantly different changes in bone structure over 12 months. Huynh-Feldt corrected F-tests will be used in order to account for any possible violation of sphericity. It is hypothesized that the Exercise group will have significantly greater improvements than the Control or Risedronate groups relative to bone structure. If there is a higher than expected attrition rate, a maximum likelihood estimation method (e.g. mixed models) will be used instead of RM-ANOVA in order to utilize all available data and not delete cases in a list wise manner.

For Aim 2, hypothesis 2a states that “*At 12 months, there will be significantly greater improvements in BMD at the total hip, femoral neck, and spine in Exercise and Risedronate subjects compared to subjects in the Control group*”. This hypothesis compares the groups’ changes in BMD and will also be analyzed using RM-ANOVA. Hypothesis 2b states that “*At least 80 % of subjects in both the Exercise and Risedronate groups will have preserved BMD at the total hip, femoral neck, and spine”.* This hypothesis assesses the proportions of preservation of BMD in each group. It is expected that at least 80 % of subjects in both the Exercise and Risedronate groups will have preserved BMD at the three skeletal sites, and that the Control/CaD group will have a significantly lower proportion of participants preserving BMD over 12 months. In order to classify individuals into preserved vs. declined groups, change in BMD over 12 months will be compared to the least significant difference as defined by the Hologic DXA manufacturer for each respective skeletal site. Differences of proportion of preservation across groups will be assessed via Chi-Square tests, which would indicate if preservation of BMD was significantly different across groups.

Aim 3 investigates the changes in bone formation and resorption over 12 months comparing AlkphaseB and Serum NTx measurements at baseline, 6, and 12 months. The relationships of interest in Aim 4 will be explored by correlations between percentage of exercise sessions attended, percentage of risedronate pills taken, and 12-month changes in bone structure at the tibia and hip (BSI and HSA respectively).

### Data and safety monitoring board

The conduct and scientific integrity of this proposed clinical trial will be monitored by a Data and Safety Monitoring Committee (DSMC), comprised of appointed, doctorally-prepared research faculty from the College of Nursing, the College of Allied Health, and the Department of Statistics. The DSMC will audit the implementation of the protocol beginning 6 months after the start of subject accrual. Since this trial is greater than minimal risk, it will be audited every 6 months. Each report will include monitoring of: compliance with informed consent and eligibility requirements, compliance with the recruitment plan according to protocol, follow-up data collection according to protocol, expected and actual accrual, protocol violations, and patient withdrawals from the study.

If a subject suffers an unanticipated serious adverse event related to this study protocol, investigators will submit a Report of an Internal Adverse Event to the Institutional Review Board (IRB), the Data and Safety Monitoring Committee (DSMC), and to our funding source (National Institute of Nursing Research) within 48 h as well as notifying the subject’s primary care provider.

### Early termination of the study

This study will be stopped if any one of the following occurs: a) less than 50 subjects are accrued for each group within the 42 month recruiting period; b) >10 % of the subjects develop serious adverse events related to use of calcium and vitamin supplements, risedronate, or exercises; or c) 50 % attrition of subjects in either group occurs including BMD T-scores falling below -2.5.

#### Timeline

This longitudinal study will take 5 years to complete. The first 6 months of the study is allotted for start-up activities. Study enrollment will begin in Year 1, month 6 and continue through Year 4, month 9. We anticipate enrolling at least 9 subjects per month during the 42 months of active enrollment. Subjects will complete the study by the 9th month of year 5 and final analysis and report writing will take place the last 3 months of the study. See Table 3.

**Table 3:** Timeline for implementation of study

TIMELINE	Year 1				Year 2				Year 3				Year 4				Year 5			
Months:	3	6	9	12	3	6	9	12	3	6	9	12	3	6	9	12	3	6	9	12
Develop training manuals, scripts, educational programs	→	→																		
Hire / orient team members	→	→																		
Finalize recruitment, intervention, data collection protocols	→	→																		
IRB approval	→	→																		
Enroll subjects			→	→	→	→	→	→	→	→	→	→	→	→	→					
Implement interventions and data collection			→	→	→	→	→	→	→	→	→	→	→	→	→	→	→	→		
Quality control checks			→	→	→	→	→	→	→	→	→	→	→	→	→	→	→	→		
Team meetings		→	→	→	→	→	→	→	→	→	→	→	→	→	→	→	→	→		
Data analysis							→	→	→	→	→	→	→	→	→	→	→	→	→	→
Final report writing																				→
Manuscript preparation									→	→	→	→	→	→	→	→	→	→	→	→

## Discussion

Osteoporosis and osteoporotic fractures are major health care problems resulting in significant morbidity and mortality for patients and significant U.S. health care costs. The following are two of the many reasons why this study is significant:

1. This study addresses the importance of treating women with low bone mass to prevent further bone loss, especially when women are in the rapid bone loss phase during their first 5 years’ post-menopause. Because of concerns about their long-term use, providers are reluctant to prescribe BPs for early post-menopausal women who have low bone mass. More studies are needed testing whether bone-loading exercise programs can replace use of BPs in preventing bone loss in these women.
2. There is a critical need for studies that have measures of bone strength in addition to BMD, such as measures of bone structure and bone turnover. Bone strength is the best predictor of fracture risk, and BMD is only one measure of bone strength. Researchers have reported that bone strength improves with exercises even when BMD scores are decreased, most likely because exercise can improve bone structure as well as BMD [12, 23].

### Controversies in management

Experts agree on the need for women with low bone mass to be diagnosed and to receive treatment to prevent further bone loss. Currently, women with low bone mass are encouraged to increase their intake of calcium and vitamin D, and to increase their physical activity, and providers may also prescribe BPs for these women. Short-term use of BPs is relatively safe and is the standard of care for women diagnosed with osteoporosis. Numerous studies have documented the effectiveness of the BP “risedronate” in prevention of bone loss and fractures. The NOF recommends that BPs be prescribed for all patients with vertebral or hip fractures, femoral neck or spine T-scores of < -2.5 (dx of osteoporosis), and a 10-year high risk probability of fracture based on results of the FRAX tool [3].

Treatment with BPs in women with low bone mass who do not meet these criteria is controversial. Even though prescriptions for BPs may not be indicated for all women with low bone mass, preventative treatment with BPs has been approved by the FDA and BPs are commonly prescribed for these women. Experts across the world warn that many women unnecessarily are prescribed BPs to prevent bone loss and fractures [41]. While short-term use of BPs is relatively safe for most women, the safety of long-term use of BPs (greater than 5 years) is unclear [15].

In 2005, reports of atypical femur fractures began to surface in women taking the BP alendronate for 5 to 10 years [42]. Atypical fractures are believed to occur because long-term use of BPs can result in a severe suppression of bone turnover with significant accumulation of micro-damage in bone [43]. This micro-damage can lead to increased fracture risk. When BPs accumulate in bones long-term, they cause bones to be “harder” but not necessarily “tougher”. In a recent study, long-term use of BPs was associated with a 20 % reduction in bone “toughness” (that is, the ability to endure bending pressure without breaking) [18].

Discontinuation of BPs after 5 years of treatment is becoming a common practice, particularly for patients at low risk of fracture. More research is needed to examine a weakening of the mechanical properties of bone as a possible side effect of BPs. The proposed research addresses concerns about long-term use of BPs by determining whether an exercise regimen with appropriate loading characteristics could replace or delay use of BPs in at-risk women.

### Critical need for measures of bone structure and turnover as well as BMD

Stronger bones are less likely to fracture. However, because BMD is only one measure of bone strength, an increase in BMD explains less than half of the observed reduction in fractures. Bones are strengthened by restoring normal architecture of bone and decreasing bone turnover as well as by increasing BMD [44]. Thus, to better understand the impact of exercise on bone strength, outcomes in the proposed study will include bone structure as well as BMD.

#### Bone structure

Compared to premenopausal women, both cortical thickness and trabecular bone volume in postmenopausal women are substantially reduced [45, 46]. Uusi-Rasi (2003) investigated the effects of weight bearing jumping exercises on BMD and bone structure in 164 postmenopausal women. Findings were that subjects who exercised had no improvement in BMD but did have improved bone structure (BSI) at some of the most heavily loaded sites [12]. Heinonen (2012) reported that an 18 month high-impact exercise intervention strengthened the femoral neck in premenopausal women by enhancing the structural properties as measured by HSA [47].

#### Bone turnover

Biomarkers of bone turnover predict rapidity of bone loss in women, may predict risk of fracture independently of bone density, and may help determine adequacy of patient adherence to treatments for bone loss [36, 48]. One remodeling cycle of resorption and formation takes up to six months with approximately 5–15 % of total bone mass replaced per year. Menopause results in a brief period (~5 years) of accelerated turnover with resorption far exceeding formation. Increased resorption will result in bone loss. With exercise, there is new bone formation in areas of mechanical strain resulting in bones with greater bending strength in those regions. While weakening mechanical properties of bone (such as decreased bending strength) are possible with long-term use of BPs (>5 years), long-term participation in targeted exercise training continually improves mechanical properties of bone over time. This provides the rationale for the comprehensive examination of bone (bone structure, BMD, and bone turnover) proposed in the study.

### Summary

Our research aims to decrease the risk of osteoporotic fractures by improving bone strength and preserving BMD in women with low bone mass during their first 5 years post-menopause, a time of rapid and significant bone loss. Our study compares effectiveness of bone-loading exercises with either risedronate or CaD alone. It is important to understand the specific effects of each treatment for maintaining bone health in women over the long-term. Bone-loading exercise programs for post-menopausal women with low bone mass may more effectively improve bone strength (structure, turnover, BMD) than either risedronate or CaD alone. Prescribing exercise programs and delaying use of BPs can prevent adverse effects from long-term use of BPs and provide the opportunity for later use when they may be critically needed. Results of this study could be used in developing a clinical management pathway for women with low bone mass at their peak period of bone loss that would involve lifestyle modifications such as exercises prior to medications such as BPs.

## Additional file

Additional file 1: Bone-Loading Exercise Program. (DOCX 92 kb)

## Abbreviations

1 RM: 1 Repetition Maximum
BMD: Bone mineral density
BMI: Body mass index
BP: Bisphosphonate
BSI: Bone Strength Index
Ca: Calcium
CaD: Calcium + vitamin D
Cr: Creatinine
CSA: Cross-sectional area
DXA: Dual Energy X-ray Absorptiometry
ET: Exercise trainer
FRAX: Fracture Risk Assessment Tool
HSA: Hip Structural Analysis
IA: Iowa
IPAQ: International Physical Activity Questionnaire
IU: International Units
mg: milligram
NE: Nebraska
ng/ml: nanograms per milliliter
NOF: National Osteoporosis Foundation
PARQ: Physical Activity Readiness Questionnaire
pQCT: Peripheral Quantitative Computed Tomography
PTH: Parathyroid hormone
RC: Research coordinator
Redcap: Research Electronic Data Capture
RET: Research exercise trainer
RM-ANOVA: Repeated Measures Analysis of Variance
serum 25(OH)D: Serum measure of vitamin D
TSH: Thyroid stimulating hormone
Z: Section modulus (measure of hip structure)

## References

[1] Wright N, Looker A, Saag K, et al. The recent prevalence of osteoporosis and low bone mass based on the bone mineral density at the femoral neck or lumbar spine in the united states. J Bone Miner Res. 2014;29 (11): 2520-26. 10.1002/jbmr.2269 PMC4757905 24771492

[2] Borgström F, Lekander I, Ivergård M (2013). The international costs and utilities related to osteoporotic fractures study (ICUROS)—quality of life during the first 4 months after fracture. Osteoporosis Int.

[3] Cosman F, De Beur S, LeBoff M (2014). Clinician’s guide to prevention and treatment of osteoporosis. Osteoporosis Int.

[4] Lee Y, Lee Y, Ha Y, Koo K (2013). Five-year relative survival of patients with osteoporotic hip fracture. J Clin Endocrinol Metab.

[5] Cauley JA (2013). Public health impact of osteoporosis. J Gerontol A Biol Sci Med Sci.

[6] Singer A, Exuzides A, Spangler L (2015). Burden of illness for osteoporotic fractures compared with other serious diseases among postmenopausal women in the united states. Mayo Clin Proc.

[7] Frisoli A, Chaves PH, Ingham SJ, Fried LP (2011). Severe osteopenia and osteoporosis, sarcopenia, and frailty status in community-dwelling older women: results from the women’s health and aging study (WHAS) II. Bone.

[8] Recker R, Lappe J, Davies K, Heaney R (2000). Characterization of perimenopausal bone loss: a prospective study. J Bone Mineral Res.

[9] Prentice R, Pettinger M, Jackson R (2013). Health risks and benefits from calcium and vitamin D supplementation: women’s health initiative clinical trial and cohort study. Osteoporosis Int.

[10] Roux C, Binkley N, Boonen S (2014). Vitamin D status and bone mineral density changes during alendronate treatment in postmenopausal osteoporosis. Calcif Tissue Int.

[11] McClung MR, Benhamou C, Man Z (2013). A novel monthly dosing regimen of risedronate for the treatment of postmenopausal osteoporosis: 2-year data. Calcif Tissue Int.

[12] Uusi-Rasi K, Kannus P, Cheng S (2003). Effect of alendronate and exercise on bone and physical performance of postmenopausal women: A randomized controlled trial. Bone.

[13] Anitha D, Kim K, Lim S, Lee T (2013). Implications of local osteoporosis on the efficacy of anti-resorptive drug treatment: A 3-year follow-up finite element study in risedronate-treated women. Osteoporosis Int.

[14] Angthong C, Angthong W (2011). Unusual subtrochanteric femoral insufficiency fractures associated with the prolonged use of alendronate and risedronate: a report of two cases. J Med Assoc Thai.

[15] Shane E, Burr D, Abrahamsen B (2014). Atypical subtrochanteric and diaphyseal femoral fractures: second report of a task force of the american society for bone and mineral research. J Bone Miner Res.

[16] Odvina CV, Zerwekh JE, Rao DS, Maalouf N, Gottschalk FA, Pak CYC (2005). Severely suppressed bone turnover: a potential complication of alendronate therapy. J Clin Endocrinol Metab.

[17] Ott SM (2005). Long-term safety of bisphosphonates. J Clin Endocrinol Metab.

[18] Stepan JJ, Burr DB, Pavo I (2007). Low bone mineral density is associated with bone microdamage accumulation in postmenopausal women with osteoporosis. Bone.

[19] Schneider J (2006). Should bisphosphonates be continued indefinitely? An unusual fracture in a healthy woman or long-term alendronate. Geriatrics.

[20] Bauer DC, Schwartz A, Palermo L (2014). Fracture prediction after discontinuation of 4 to 5 years of alendronate therapy: the FLEX study. JAMA Intern Med.

[21] Lenard L. Bisphosphonates: Bone strengtheners or bone hardeners? Townsend Letter: The Examiner of Alternative Medicine. 2009;312:76–81.

[22] Martyn-St James M, Carroll S (2009). A meta-analysis of impact exercise on postmenopausal bone loss: the case for mixed loading exercise programmes. Br J Sports Med.

[23] Kido S, Kuriwaka-Kido R, Umino-Miyatani Y (2010). Mechanical stress activates smad pathway through PKCdelta to enhance interleukin-11 gene transcription in osteoblasts. PLoS One.

[24] Kemmler W, Engelke K, Lauber D, Weineck J, Hensen J, Kalender WA (2002). Exercise effects on fitness and bone mineral density in early postmenopausal women: 1-year EFOPS results. Med Sci Sports Exerc.

[25] Linnebur SA, Vondracek SF, Vande Griend JP, Ruscin JM, McDermott MT (2007). Prevalence of vitamin D insufficiency in elderly ambulatory outpatients in denver, colorado. Am J Geriatr Pharmacother.

[26] Lappe JM, Davies KM, Travers-Gustafson D, Heaney RP (2006). Vitamin D status in a rural postmenopausal female population. J Am Coll Nutr.

[27] Vieth R, Bischoff-Ferrari H, Boucher BJ (2007). The urgent need to recommend an intake of vitamin D that is effective. Am J Clin Nutr.

[28] Waltman NL, Twiss JJ, Ott CD (2010). The effect of weight training on bone mineral density and bone turnover in postmenopausal breast cancer survivors with bone loss: a 24-month randomized controlled trial. Osteoporos Int.

[29] Fogelman I, Ribot C, Smith R, Ethgen D, Sod E, Reginster JY (2000). Risedronate reverses bone loss in postmenopausal women with low bone mass: Results from a multinational, double-blind, placebo-controlled trial. BMD-MN study group. J Clin Endocrinol Metab.

[30] Peppone LJ, Mustian KM, Janelsins MC (2010). Effects of a structured weight-bearing exercise program on bone metabolism among breast cancer survivors: a feasibility trial. Clin Breast Cancer.

[31] Courneya KS, Friedenreich CM, Sela RA, Quinney HA, Rhodes RE, Jones LW (2004). Exercise motivation and adherence in cancer survivors after participation in a randomized controlled trial: an attribution theory perspective. Int J Behav Med.

[32] Knobf MT, Insogna K, DiPietro L, Fennie K, Thompson AS (2008). An aerobic weight-loaded pilot exercise intervention for breast cancer survivors: bone remodeling and body composition outcomes. Biol Res Nurs.

[33] Bandura A (1997). Self-efficacy: The exercise of control.

[34] Rogers L, Markwell S, Hopkins-Price P (2011). Reduced barriers mediated physical activity maintenance among breast cancer survivors. J Sport Exerc Psychol.

[35] Rogers L, Fogleman A, Verhulst S, Malone J, Robbs R, Robbins K. Refining measurement of social cognitive theory factors associated with exercise adherence in head & neck patients. J Psychosoc Oncol. 2015;33(5):467–87. 10.1080/07347332.2015.1067277 26177345

[36] Burch J, Rice S, Yang H (2014). Systematic review of the use of bone turnover markers for monitoring the response to osteoporosis treatment: the secondary prevention of fractures, and primary prevention of fractures in high-risk groups. Health Technol Assess.

[37] Bonnick SL (2013). Dual-energy x-ray absorptiometry: interpreting reports and serial measurements. Clin Obstet Gynecol.

[38] McCloskey EV, Odén A, Harvey NC (2015). Adjusting fracture probability by trabecular bone score. Calcif Tissue Int.

[39] Ohnaru K, Sone T, Tanaka K, et al. Hip structural analysis: A comparison of DXA with CT in postmenopausal japanese women. Springerplus. 2013;2:331-1801-2-331. eCollection 2013. 10.1186/2193-1801-2-331 PMC3724983 23961402

[40] Garnero P (2006). Biochemical markers of bone turnover.

[41] Pate D (2010). Long-term bisphosphonate use and increased fracture risk. Dynamic Chiropract.

[42] Kennel KA, Drake MT (2009). Adverse effects of bisphosphonates: Implications for osteoporosis management. Mayo Clin Proc.

[43] Mashiba T, Turner CH, Hirano T (2001). Effects of high-dose etidronate treatment on microdamage accumulation and biomechanical properties in beagle bone before occurrence of spontaneous fractures. Bone.

[44] van der Linden JC, Weinans H (2007). Effects of microarchitecture on bone strength. Curr Osteoporos Rep.

[45] Akhter MP, Lappe JM, Davies KM, Recker RR (2007). Transmenopausal changes in the trabecular bone structure. Bone.

[46] Vainionpaa A, Korpelainen R, Sievanen H, Vihriala E, Leppaluoto J, Jamsa T (2007). Effect of impact exercise and its intensity on bone geometry at weight-bearing tibia and femur. Bone.

[47] Heinonen A, Mäntynen J, Kannus P (2012). Effects of high-impact training and detraining on femoral neck structure in premenopausal women: a hip structural analysis of an 18-month randomized controlled exercise intervention with 3.5-year follow-up. Physiother Can.

[48] Eastell R, Bainbridge P, Orwoll ES, Bliziotes M (2003). Bone turnover markers: Their place in the investigation of osteoporsis. Osteoporosis: Pathophysiology and clinical management.

