# Supplementary material for: Protocol for a randomized controlled trial to compare bone-loading exercises with risedronate for preventing bone loss in osteopenic postmenopausal women
Source: BMC Womens Health. 2016 Aug 30;16(1):59. doi: 10.1186/s12905-016-0339-x (PMC5004254; doi:10.1186/s12905-016-0339-x)
Supplement: Additional file 1: — Bone-Loading Exercise Program. (DOCX 92 kb) [file 12905_2016_339_MOESM1_ESM.docx]

**Appendix A. Bone-Loading Exercise Program**

Exercise sessions will occur three times per week at partner community fitness centers. Each session will include warm up and cool down exercises consisting of five minutes of slow walking with at least one day of rest required between each study exercise session.

**Resistance Training**

Participants will alternate between muscle groups exercised and allow two minutes rest between consecutive sets of an individual exercise. Months 1-6 resistance training on machines include overhead press, chest press, latissimus pull, seated row, leg press, and hamstring curl. Months 7-12 resistance training on machines include overhead press, chest press, latissimus pull, seated row, leg press, and hamstring curl. Progression protocol for these resistance exercises is outlined in Table 4.


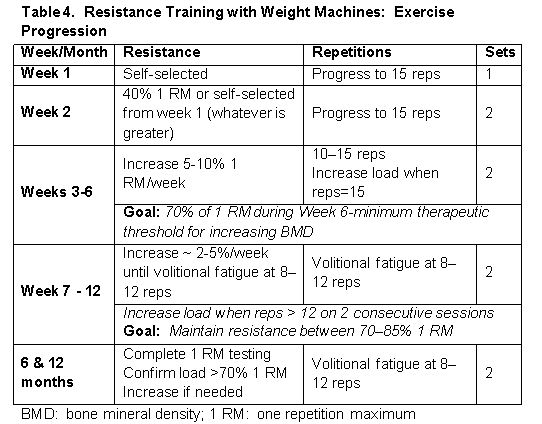


Progression will be prescribed based on the participant’s estimated 1 repetition maximum (RM), participant tolerance, and the progression tables. Resistance will be based on the estimated 1 RM performed on the chest press and leg press machines (with a goal of reaching a resistance > 70% of 1 RM). Resistance for all other exercises will be gradually increased to a load that results in volitional fatigue at 8-12 repetitions by week 7. The protocol incorporates slow progression to improve tolerance and minimize soreness and risk of injury. Strength assessment (estimated 1RM) occurs in week 2 to minimize risk of injury. Reassessment of 1 RM will occur at 6 months in order to validate that the intervention is resulting in strength increases and to evaluate if the workload is at a therapeutic potential for bone (goal: workload > 70% of 1RM).

For those exercises using body weight for resistance, load will be added using a weighted vest, progressed exercise movement, or increased hold time. Both resistance and repetitions will be increased over time according to participant tolerance. The progression protocol is outlined in Table 5. During months 1-6 body weight exercises include step ups, quadruped superman, side bridges, and supine abdominal stabilization. During months 7-12, body weight exercises include forward lunges, supine bridges, side bridges, and abdominal stabilization.

| **Table 5. Body Weight Resistance Training:    Exercise Progression** | | | |
| --- | --- | --- | --- |
| **Week/Month** | **Resistance*** | **Sets** | **Reps*** |
| Weeks 1-2 | 0% BW | 1 | 5-12 |
| Weeks 3-4 | 0% BW | 2 | 8-12 |
| Month 2 | 1-2% BW | 2 | 8-12 |
| Month 3 | 2-5% BW | 2 | 8-12 |
| Month 4-12 | 5-10% BW | 2 | 8-12 |
| Increase load (% BW) when reps >12 on 2 consecutive sessions | | | |
| *BW=Body Weight; Reps=Repetitions | | | |

**Impact Exercise-Jogging.**

In addition to resistance exercise, the exercise intervention will include high impact, weight bearing exercises with high ground reaction forces to promote bone remodeling. To accomplish this, participants will walk around a track, or any other open space, interspersed with short periods of jogging. After walking for 20-30 seconds, participants will jog the prescribed number of steps and then return to walking. To avoid working at a vigorous intensity, participants will continue walking until they reach an RPE at or below 11 on the Borg RPE scale before they begin their next set of prescribed steps. The exercise will be progressed by increasing the number of sets, steps and bodyweight. The progression protocol is outlined in Table 6.

| **Table 6. Impact Jogging Progression** | | | |
| --- | --- | --- | --- |
| **Week/Month** | **Resistance*** | **Sets** | **Steps*** |
| Weeks 1-2 | 0% BW | 3-5 | 4-6 |
| Weeks 3-4 | 0% BW | 5-10 | 6 |
| Month 2 | 0% BW | 7-10 | 6-10 |
| Month 3 | 1-3% BW | 10 | 6-10 |
| Month 4-5 | 3-5% BW | 10 | 6-10 |
| Month 6-8 | 5-7% BW | 10 | 6-10 |
| Month 9-12 | 7-10% BW | 10 | 6-10 |
| *BW=Body Weight; Steps=Steps Each Leg | | | |

**Alterations for Participants with Total Joints of the Lower Extremity**

To avoid contraindicated activity or movements, women with total joint arthroplasty of the knee or hip will perform a slightly altered exercise program. Leg press is excluded from the protocol, and step ups on an 8” platform replaces impact jogging. Goals for step up progression mirrors those for jogging.
